# Targeting Internalized *Staphylococcus aureus* Using Vancomycin-Loaded Nanoparticles to Treat Recurrent Bloodstream Infections

**DOI:** 10.3390/antibiotics10050581

**Published:** 2021-05-14

**Authors:** Danielle Nader, Fajer Yousef, Nicola Kavanagh, Benedict K. Ryan, Steven W. Kerrigan

**Affiliations:** 1 Cardiovascular Infection Research Group, School of Pharmacy and Biomolecular Sciences, RCSI University of Medicine and Health Sciences, 123 St. Stephens Green, Dublin 2, Ireland; fajeryousef92@gmail.com (F.Y.); nicolakavanagh12@gmail.com (N.K.); 2 School of Pharmacy and Biomolecular Sciences, RCSI University of Medicine and Health Sciences, 123 St. Stephens Green, Dublin 2, Ireland; benedictryan@rcsi.ie

**Keywords:** sepsis, internalization, human endothelial cell, *Staphylococcus aureus*, infection, nanoparticle, vancomycin

## Abstract

The bacterial pathogen *Staphylococcus aureus* is a leading cause of bloodstream infections, where patients often suffer from relapse despite antibiotic therapy. Traditional anti-staphylococcal drugs display reduced effectivity against internalised bacteria, but nanoparticles conjugated with antibiotics can overcome these challenges. In the present study, we aimed to characterise the internalisation and re-emergence of *S. aureus* from human endothelial cells and construct a new formulation of nanoparticles that target intracellular bacteria. Using an in vitro infection model, we demonstrated that *S. aureus* invades and persists within endothelial cells, mediated through bacterial extracellular surface adhesion, Fibronectin-binding protein A/B. After internalising, *S. aureus* localises to vacuoles as determined by transmission electron microscopy. Viable *S. aureus* emerges from endothelial cells after 48 h, supporting the notion that intracellular persistence contributes to infection relapses during bloodstream infections. Poly lactic-co-glycolic acid nanoparticles were formulated using a water-in-oil double emulsion method, which loaded 10% vancomycin HCl with 82.85% ± 12 encapsulation efficiency. These non-toxic nanoparticles were successfully taken up by cells and demonstrated a biphasic controlled release of 91 ± 4% vancomycin. They significantly reduced *S. aureus* intracellular growth within infected endothelial cells, which suggests future potential applications for targeting internalised bacteria and reducing mortality associated with bacteraemia.

## 1. Introduction

*Staphylococcus aureus* is a universal Gram positive bacteria that lives harmlessly on the skin and mucous membranes. It can cause superficial skin lesions and wound infections as well as more serious debilitating invasive diseases such as sepsis [1]. The current principles of sepsis management are to optimise organ perfusion with intravenous fluids, support dysfunctional organ systems (e.g., ventilation) and mitigate the immediate threat of uncontrolled infection with antibiotic therapy and source control. With significant advances in sepsis recognition and supportive care, the in-hospital mortality rate has improved over time and this has resulted in a significant increase in the number of sepsis survivors. The greatest concern for those who survive sepsis is increased risk of recurrent infections. Current evidence suggests that between 30 and 42% of sepsis survivors relapse within the first 90 days after being discharged from hospital [2,3,4]. Treatment failure is defined as persistence of infection following 10 days after therapy started, recurrence of infection within 60 days of discontinuation of therapy or death in a 30-day period post therapy.

The vascular endothelium is a major target of sepsis-induced events and a number of pathogens, including *S. aureus*, have been shown to bind to and internalize into human vascular endothelial cells [5,6]. The process of internalization is mediated by the major *S. aureus* cell wall proteins, fibronectin binding proteins A/B (FnbpA/B) which bind plasma fibronectin and cross link to the integrin α5β1 [7]. Integrin engagement triggers bacterial internalization in a process driven by endothelial cell actin remodelling, focal adhesion kinase and Src family kinases [8]. Once internalized, neither immune cells nor antibiotics can reach the bacteria, rendering them safe from host immunity and circulating anti-staphylococcal antibiotics, thus encouraging its survival and persistence. Infection recurrence occurs as a result of the re-emergence of these internalized bacteria back into the bloodstream at a later time point. Although sepsis is often treated with antibiotic cocktails composed of anti-staphylococcal compounds including flucloxacillin, teicoplanin, clindamycin and linezolid, research has shown they are unable to kill internalised bacteria since these antibiotics have poor cell penetration [9,10]. Therefore, relapse may occur due to the lack of access of the antibiotics to the intracellular bacteria, suggesting that bacterial invasion of endothelial cells by *S. aureus* is a pathogenic mechanism used to evade antibiotic treatment and prolong infection.

The growing concern around the lack of treatment for sepsis coupled with the antimicrobial resistance crisis places an immense pressure on alleviating the mortality and morbidity associated with this pathogen. Traditional antimicrobials exhibit limitations in clinical settings, displaying minimal permeation to the infection nidus [11,12,13]. However, the delivery of drugs using nanoparticles has profoundly altered the pharmaceutical landscape and can circumvent the issues associated with traditional antibiotic delivery. By fine-tuning its physiochemical properties such as size, surface charge, material composition, and drug release, nanoparticles can be optimised for specific purposes and significantly improve the efficacy of antibiotic treatment. Largely attractive aspects of nanoparticles are its small diameter (10–500 nm), high surface-to-volume ratio, capacity to encapsulate various drug compounds, sustained drug release, and controlled delivery [14]. Whilst conventional antibiotics display poor host membrane permeability, nanoparticles can transport drugs via endocytosis which facilitates intracellular targeting. This enhances bacteria eradication and improves efficacy of the antibacterial drug. Nanoparticles can be even further customised by using various coatings including polymers, lipids, liposomes, inorganic metals, or silica which alters their biocompatibility and clearance by the reticuloendothelial system. Additionally, these surfaces can be designed with ligands to increase affinity to host cells or bacterial surfaces to encourage active targeting [11]. Loading antibiotics into nanoparticles have displayed significant promise towards improving drug delivery and in vitro antimicrobial activity amongst notably harmful microbes, including *S. aureus*, *Escherichia coli*, and *Pseudomonas aeruginosa* [15,16,17]. Overall, coupling traditional antibiotics with drug-encapsulated nanoparticles offer clear potential for a delivery system that could be exceedingly useful in addressing the limitations with conventional antibiotic treatments.

Using an ex vivo dynamic model of human endothelial cells, we demonstrated that *S. aureus* was capable of binding to and re-emerging from human vascular endothelial cells via fibronectin binding proteins, thus evading immune and antibiotic attack. We also demonstrated the internalisation of *S. aureus* from host cells and constructed a new formulation of vancomycin-loaded nanoparticles which targeted the internalized bacterium to prevent recurrent infection. These results have profound implications for the treatment and management of recurrent infection in sepsis patients.

## 2. Results

### 2.1. Staphylococcus aureus Internalises into Human Endothelial Cells Using Fibronectin Binding Protein and Re-Emerges to Cause Infection

**Figure 1:**
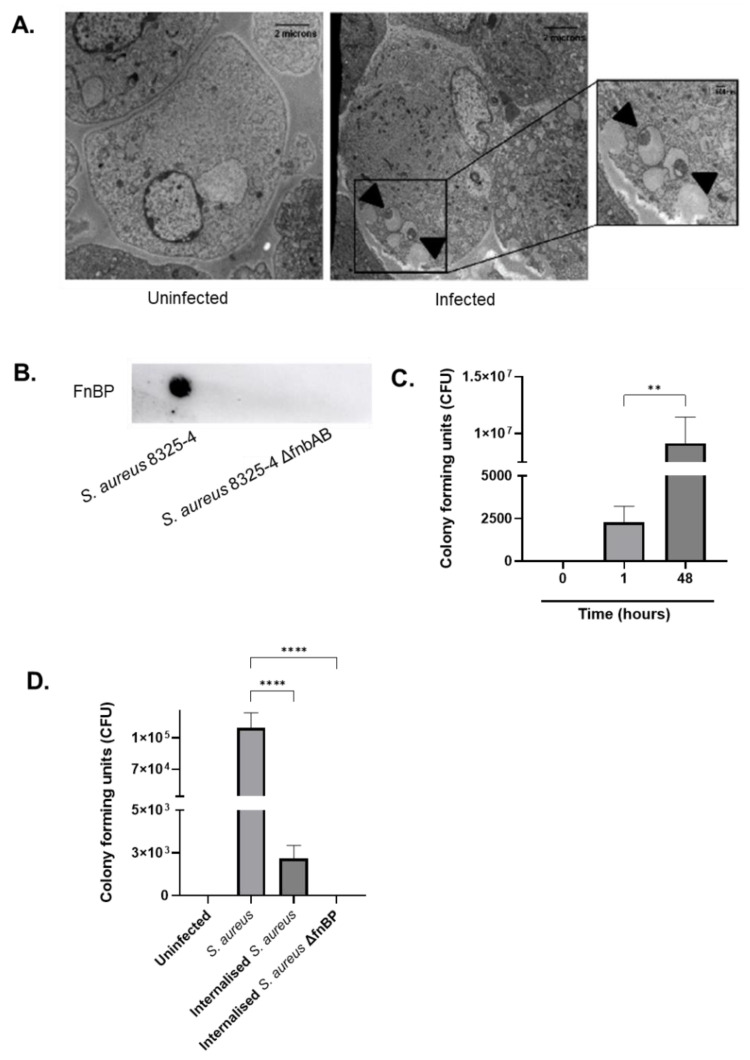
*Staphylococcus aureus* internalises into endothelial cells via its surface fibronectin binding proteins. (**A**) Transmission electron microscopy images of uninfected and *S. aureus* 8325-4 infected endothelial cells after 48 h were visualised. The side panel demonstrates the internalisation of *S. aureus* which localises to membrane vacuoles. Scale bars of uninfected and infected at 2 µm. Scale bars of side panel at 500 nm. (**B**) Dot blot analysis showing FnBP is expressed only in wild-type strain *S. aureus* 8325-4, and not in FnBP knockout strain *S. aureus* 8325-4 ΔfnbAB. (**C**) Endothelial cells were infected with *S. aureus* 8325-4 over 48 h and intracellular bacteria were quantified as colony forming units, where significant increase in growth was observed (*p* < 0.01). (**D**) Primary derived human aortic endothelial cells were infected with the wild-type *S. aureus* 8325-4 or the mutant *S. aureus* 8325-4 ΔfnbAB for 1 h. Cells were lysed and total bacteria (external and internal) were quantified using colony forming units. The experiment was repeating using the gentamycin assay method by treating endothelial cells with 100 µg/mL gentamicin HCl for 1 h. Cells were lysed and bacteria were quantified similarly. Wild-type *S. aureus* significantly internalised (*p* < 0.0001), yet the strain lacking the fibronectin binding proteins was unable to internalise into the endothelial cells. Results are the mean values ± SEM of three independent experiments using 1-way ANOVA followed by Tukey’s post hoc tests. *p* < 0.05 indicates statistical significance. ** *p* < 0.01, **** *p*< 0.0001.

We first aimed to characterise the intracellular persistence of *S. aureus* 8325-4 since relapse from infections constitute a major sepsis-related issue. To identify whether *S. aureus* was capable of internalising into human endothelial cells, transmission electron microscopy was performed. Image analysis revealed that *S. aureus* successfully enters endothelial cells during the first hour of infection and resides in the vacuoles (Figure 1A). To confirm that *S. aureus* utilises fibronectin binding proteins for entry into host cells, we used an isogenic mutant derived from the wild-type strain 8325-4 which lacks expression of these surface proteins. A dot plot confirmed this, and therefore this strain was used to further investigate the behaviour of intracellular bacteria (Figure 1B). A 48 h time course model using primary derived human endothelial cells was performed and revealed the number of viable intracellular bacteria showed a stark increase following 1 h post-infection, then progressively escalated across 48 h (Figure 1C). Next, cells were infected with both wild-type and knockout *S. aureus* strains. The cells were sheared to mimic the haemodynamic forces experienced within the vasculature in vivo, and then subject to a gentamicin protection assay. This method utilises a widely employed antibiotic renowned for its inability to penetrate eukaryotic cells and poor effectivity against *S. aureus*, therefore making it ideal for investigating invasion potential. After 1 h, *S. aureus* 8325-4 was capable of internalising into host cells, whereas the isogenic mutant was unable to penetrate (Figure 1D).

### 2.2. Optimising Vancomycin HCl Drug-Loaded Nanoparticle Characteristics

**Figure 2:**
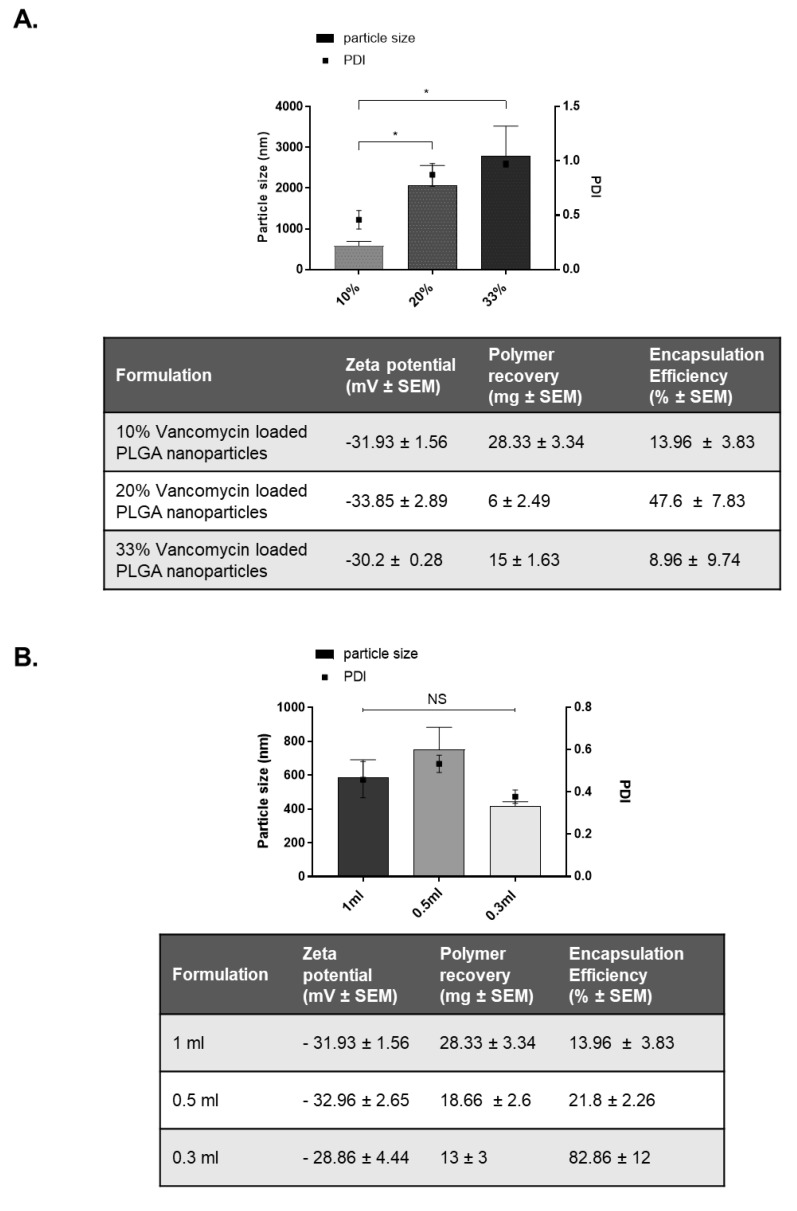

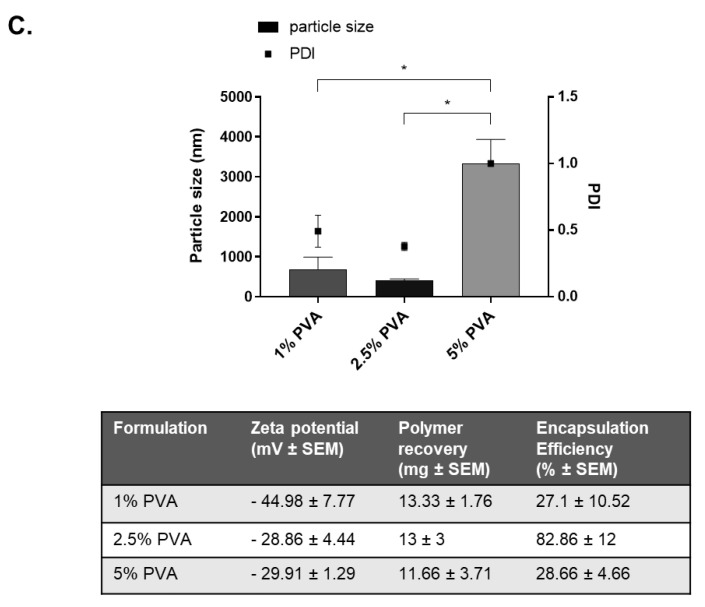
Physiochemical characteristics of vancomcyin-loaded PLGA RG 503H nanoparticles. (**A**) The effect of drug:polymer ratio was evaluated using 10%, 20% and 33% *w*/*w* of vancomycin HCl. Particle sizes were significantly different between 10% and 20% vancomycin, and 10% and 33% vancomycin (*p* < 0.05). The encapsulation efficiency was highest for the 20% formulation; however, considering that small particle sizes are most favourable for nanoparticle use, the 10% vancomycin drug:polymer ratio was chosen for future experiments. (**B**) The effects of altering the volume of the inner aqueous phase on 10% *w*/*w* vancomycin nanoparticles was investigated using 1 mL, 0.5 mL, and 0.3 mL. Reducing the inner aqueous volume did not have a significant difference on average particle size diameter or PDI. However, batches using inner aqueous phase volume of 0.3 mL had the highest encapsulation efficiency of vancomycin and therefore were used for future experiments. (**C**) The effect of changing the concentration of the surfactant polyvinyl alcohol (PVA) was assessed on 10% *w*/*w* vancomycin nanoparticles. Formulations with 5% PVA produced unwanted large particle sizes in contrast to both 1% and 2.5% (*p* < 0.05). Whilst PVA concentrations had no significant effect on polymer recovery, using 2.5% PVA produced highest encapsulation efficiency and were used for future experiments. Results are the mean values ± SEM of three independent experiments using 1-way ANOVA followed by Tukey’s post hoc tests. *p* < 0.05 indicates statistical significance. * *p* < 0.05.

A double emulsion method was used to encapsulate Vancomycin HCl into blank PLGA nanoparticles. To optimise intracellular uptake, stability, and encapsulation efficiencies of these formulations, various formulation parameters were examined. A base formulation as described previously was formulated first. The effect of alterations in Drug:Polymer ratios that equate to 10% (Base formulation), 20%, and 33% *w*/*w* Vancomycin HCl were evaluated (Figure 2A). Increasing the drug concentrations led to a significant increase in particle size diameter which ultimately resulted in the development of microparticles outside of the desired nanoparticle size range (<1000 nm). These data evidently demonstrate that increasing the drug content is disadvantageous to maintaining the desired nanometre size range. Moreover, as the particle size increases, the PDI of the formulation increased, which was indicative of a polydisperse sample with multiple sub-populations. Considering both size and PDI ranges, it was concluded that the 20% *w*/*w* and 33% *w*/*w* formulations were unsuitable for Vancomycin encapsulation. Therefore, the 10% *w*/*w* sample was selected for further investigation. Another challenge experienced when formulating these particles was that Vancomycin HCl is a hydrophilic moiety which tends to partition into the aqueous phase during development. By altering the volume of the inner aqueous phase, the encapsulation efficiency of vancomycin PLGA nanoparticles can be maximised (Figure 2B). Modifying this step using 0.3 mL to 1 mL did not significantly affect the particle size or zeta potential of the formulations. However, reducing the volume of the aqueous phase enhanced the encapsulation efficiency of vancomycin. Therefore, using 10% *w*/*w* vancomycin-loaded PLGA nanoparticles, 0.3 mL of inner aqueous volume showed the highest encapsulation efficiency of 82.8 ± 12% and was chosen for further investigation. Finally, surfactants play a critical role in regulating final particle size through stabilization of the oil–water emulsion step. Polymeric surfactants such as polyvinyl alcohol (PVA) may affect encapsulation efficiencies due to their strong absorption properties and therefore may aid in preventing the release of core contents within the nanoparticle. We evaluated the effect of changes to surfactant concentration on 10% *w*/*w* vancomycin-loaded PLGA nanoparticles. Altering PVA concentration between 1% and 5% resulted in significant changes to size and PDI. The latter resulted in undesirable large particle diameters and high PDI, indicative of nanoparticles that were outside of the acceptable size range with multiple subpopulations. Therefore, the use of 2.5% *w*/*v* PVA resulted in the most optimal encapsulation efficiency at 82.8 ± 12% (Figure 2C).

### 2.3. Rhodamine B-Loaded PLGA Nanoparticles Internalise into Cells

Rhodamine B-loaded PLGA nanoparticles were used to assess the ability of the double emulsion solvent evaporation method to formulate nanoparticles with the necessary characteristics to internalise into cells. Formulated nanoparticles showed negative zeta potentials (−23.65 ± 0.95 mV); mean ± SEM, acceptable size diameter and PDI values (477.35 ± 30.95nm; 0.3035 ± 0.0065, mean ± SEM) Given that this compound is a chemical dye that functions as a water-tracer fluorescent, its encapsulation was confirmed qualitatively.

**Figure 3:**
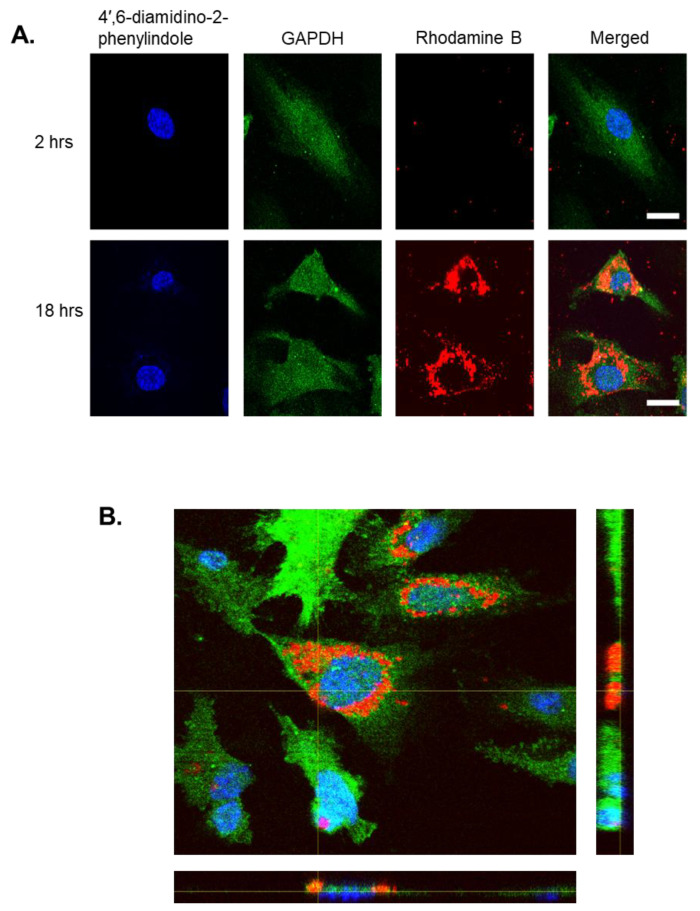
Rhodamine B-loaded PLGA RG-503H nanoparticles internalise into human endothelial cells. (**A**) Human endothelial cells were incubated over 18 hrs (hours) with Rhodamine B-loaded PLGA RG-503H nanoparticles to assess their ability to internalise. Cells were labelled with a GAPDH 14C10 monoclonal antibody for cytoplasm staining, 4′,6-diamidino-2-phenylindole for nucleus staining and Rhodamine B fluorescent dye for nanoparticle staining. Nanoparticles were found to accumulate within the cytoplasm after 18 hrs. Cells were visualised using Zeiss Axio Observer Z1 immunofluorescence microscope. Representative images of each channel alongside a merged image are shown with scale bar = 10 µM. (**B**) Z-stack images showing localisation of nanoparticles within endothelial cells. Both angles confirm nanoparticles have internalised and do not aggregate or clump on the cell exterior.

Immunofluorescence images reveal that 2 h after incubation, nanoparticles remained external to cells, whereas after 18 h the particles successfully internalised and accumulated within the cystol. This signifies that nanoparticle internalisation is a time-dependent process (Figure 3A). To visualise the localisation of Rhodamine B PLGA particles within the endothelial cells, a z-stack image was created. This confirmed that the nanoparticles were not aggregated or clumping onto the surface of the monolayer and were taken up by the host cells (Figure 3B).

Blank nanoparticles were prepared using the base formulation parameters in three independent batches with average size of 339 ± 12 nm. The average PDI was less than 0.3 and therefore the samples were considered to be monodispersed. Largely negative zeta potentials (−31.73 ± 2.4 mV) were obtained in all batches which highly favour nanoparticle stability by preventing particle aggregation.

**Figure 4:**
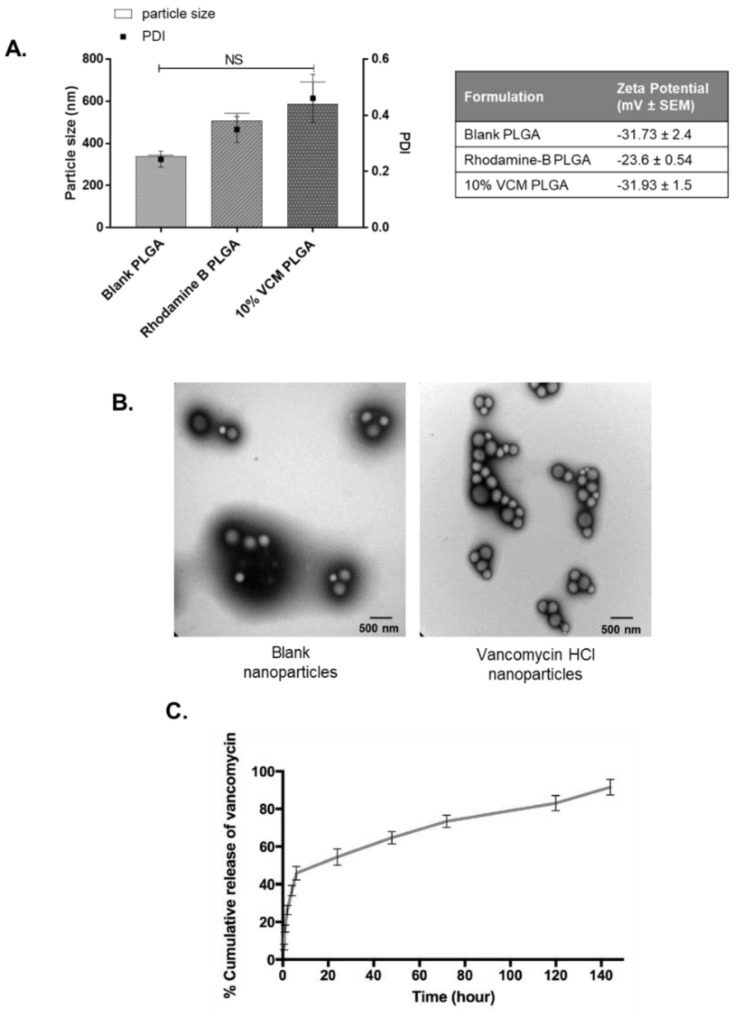
Comparative analyses of formulated nanoparticles. (**A**) The blank, Rhodamine B-, and 10% vancomycin HCl-loaded nanoparticles were cross-examined to assess the effect of drug encapsulation on particle size, PDI, and zeta potential. There was no significant difference in particle size or PDI between the nanoparticles, and all 3 nanoparticles displayed negative zeta potentials and even size distributions. (**B**) Transmission electron images of blank (left) and vancomycin HCl- (right) loaded nanoparticles indicate the heterogeneity of each particle size, all displaying smooth spherical shapes. Scale bars at 500 nm. (**C**) An n vitro drug release profile was calculated using a PUR-A-LYZER mini 6000 dialysis kit. This indicates cumulative percentage increase in vancomycin from nanoparticles over 144 hrs. Results are the mean values ± SEM of three independent experiments using 1-way ANOVA followed by Tukey’s post hoc tests.

Properties of the blank PLGA RG 503H and drug-loaded PLGA RG 503H were then compared to assess the effect of drug encapsulation on particle size, PDI and zeta potential. Rhodamine B or Vancomycin HCl PLGA RG 503H nanoparticles did not statistically affect the average diameter or PDI. Although the blank nanoparticles exhibit smaller particle sizes compared to Rhodamine B- and Vancomycin-loaded (339.6 ± 5.6 nm; 507.7 ± 35.2 nm; 589.1 ± 102.2 nm, respectively), all samples fell within the acceptable size range for nanoparticles (Figure 4A). TEM images of the blank and vancomycin-loaded PLGA nanoparticles displayed the formulated particle sizes, which confirmed they all shared similar morphology and were not affected by drug encapsulation (Figure 4B). Furthermore, the subsequent drug release profile was determined by a dialysis method in phosphate buffer at pH = 7.4 to quantify the cumulative release of vancomycin HCl from the nanoparticles over a time course of 6 days. The biphasic drug release indicates an initial rapid burst of released vancomycin within the first 6 h, followed by a slow plateau over the remaining 134 h (Figure 4C). In addition, the total cumulative amount of vancomycin released from PLGA nanoparticles was calculated to be approximately 91 ± 4% (Figure 4C).

### 2.4. Effect of Vancomycin HCl PLGA Nanoparticles on Internalised Bacteria

**Figure 5:**
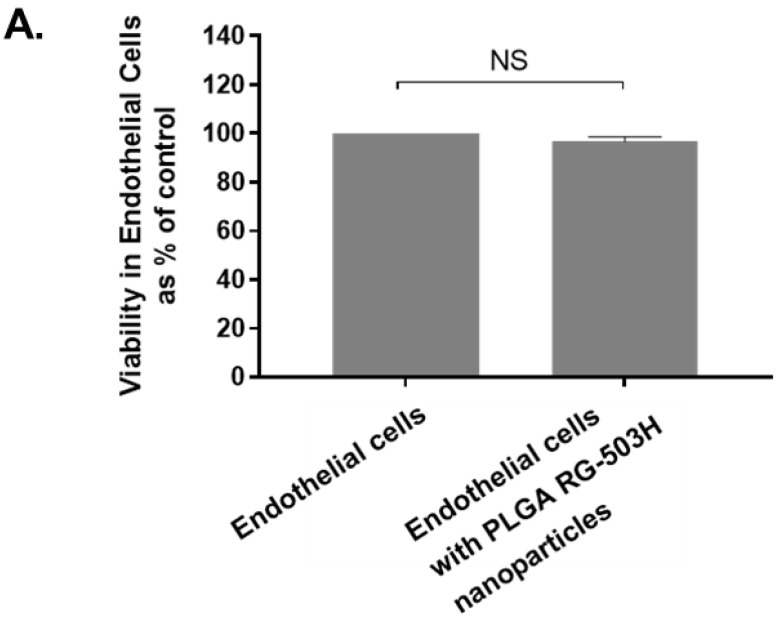

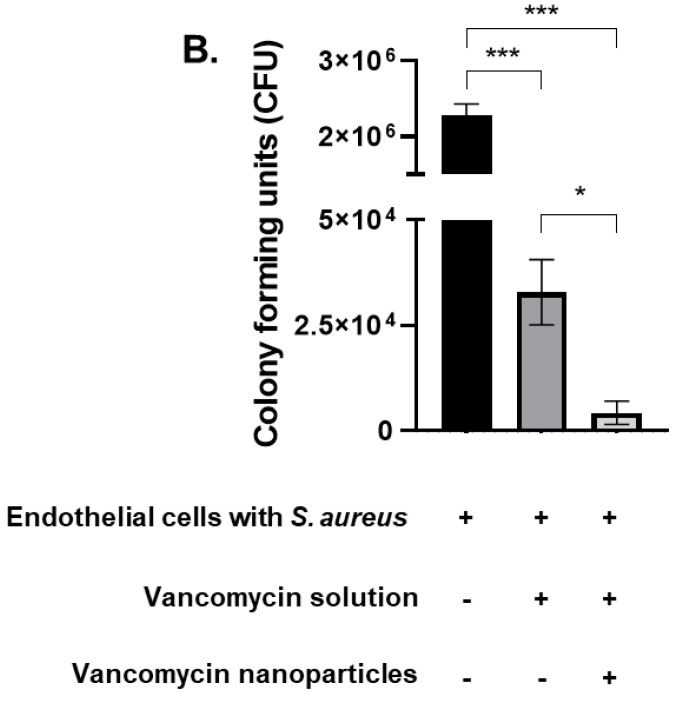
Effectivity of PLGA RG-503H nanoparticles on infected human endothelial cells in vitro. (**A**) MTT assay was performed to assess toxicity of PLGA RG-503H nanoparticles on human aortic endothelial cells. No significant difference was observed between untreated and treated cells after exposure for 24 h. (**B**) *S. aureus* 8325-4-infected endothelial cells were treated with either concentrated vancomycin solution (0.5 mg/mL), or both vancomycin solution and 10% vancomycin-loaded nanoparticles (0.5 µg/mL). Cells were lysed, and the total amount of external and internal *S. aureus* was quantified then expressed as colony forming units. The control group not exposed to any antibiotics displayed significant bacterial growth. Cells only treated with vancomycin solution were capable of reducing total *S. aureus* growth (*p =* 0.0003). Notably, cells treated with both concentrated and encapsulated vancomycin were capable of significantly reducing total *S. aureus* levels even more so than cells only treated with vancomycin solution (*p =* 0.045). Results are the mean values ± SEM of three independent experiments using 1-way ANOVA assuming unequal variances followed by Tukey’s post hoc tests. *p* < 0.05 indicates statistical significance. * *p* < 0.05, *** *p* < 0.001.

The cytotoxic effect of the blank nanoparticles was evaluated using an MTT assay that measured the metabolic activity of the human endothelial cells after 24 h incubation with 0.5 mg/mL PLGA RG 503H nanoparticles. No significant differences were observed in cell viability which demonstrates the safety of these formulations for use as a drug delivery platform (Figure 5A).

Following the confirmation of drug release and lack of cytotoxicity due to the nanoparticles, the vancomycin-loaded nanoparticles were evaluated for their ability to target internalised bacteria. Human endothelial cells were incubated with *S. aureus* 8325-4 for 24 h. External and internal *S. aureus* were quantified using methods described previously. Cells were either untreated, treated solely with a vancomycin HCl solution (0.5 mg/mL), or treated with both vancomycin solution and 10% *w*/*w* vancomycin HCl-loaded PLGA RG 503H nanoparticles (0.5 µg/mL). Infected cells that were not exposed to any antibiotic experienced significant bacterial growth, whereas both vancomycin solutions and vancomycin nanoparticles were capable of reducing *S. aureus* infections (Figure 5B).

## 3. Discussion

Morbidity and mortality associated with bloodstream infections remain significantly high, particularly in patients who concomitantly develop sepsis or acute infective endocarditis. A combination therapy of vancomycin and gentamicin is appropriate for severe cases of infective endocarditis involving persistent bacteria, due to its ability to clear extracellularly bound microbes [18]. Yet, treatment of intracellular bacteria using common antimicrobials are often ineffective. Gentamicin is unable to penetrate eukaryotic host cells. Rifampicin has demonstrated efficacy against intracellular microbes, but antibacterial resistance may render it soon unusable. Vancomycin, although remaining the initial choice for highly resistant bacteria, is poorly absorbed when administered orally or through the intestinal tract and therefore must be administered intravenously [19]. Therefore, intracellular bacteria clearly represent a significant threat during bloodstream infections and the ability of *S. aureus* to persist within host cells and later re-emerge can drastically contribute to recurrent infections experienced by some patients [20]. Within the first hour of infection, we showed *S. aureus* was capable of entering human endothelial cells through the major surface cell wall protein, FnbpA/B. This virulence factor has been demonstrated to aid in its adhesion and subsequent internalisation to host cells by binding to α5β1 integrins widely expressed across a variety of tissues and cells [7]. This contributes to internal persistence within the host cell for over 48 h, followed by re-emergence. As demonstrated by microscopy, *S. aureus* relocated to vacuoles within the endothelial cell. Rollin et al. demonstrated that *S. aureus* was able to remain within endothelial cells even after 10 days post-infection, where most infected cells were incapable of restricting bacterial growth and were lysed after 48 h. However, in other cells, bacteria resided for at least 7 days without proliferating and later escaped using an unknown pathway [21]. Synonymous with this result, we demonstrated that, in the absence of antibiotics, internalised bacteria were capable of re-emerging and remained viable, therefore rendering them infectious and capable of targeting other neighbouring cells within the monolayer. This suggests that the population of *S. aureus* that was able to survive within the host cell resulted in the formation of small colony variants (SCV) [22]. These are a slow-growing subpopulation of highly pathogenic bacteria capable of growing in externally stressful environments and contribute significantly to relapsing infections [23]. Although the mechanism by which such bacteria are capable of evading cells remains unknown, our data suggest they could be responsible for high relapse rates observed during infective endocarditis and sepsis [4].

Additionally, traditional antibiotics pose numerous challenges in modern medicine, including low bioavailability and lack of permeation to the infection niche, which is a significant limitation for the treatment of biofilms or intracellular microbes. Recently, new strategies have been employed to adjust to these complications, where the development of nanomedicine for targeted therapy against bacterial infections has been highlighted as a promising solution [24]. In the present study, we proposed a new formulation of PLGA RG 503H nanoparticles loaded with vancomycin HCl to target internalised *S. aureus* and potentially eliminate the threat of recurrent infections. Although several delivery systems for vancomycin have been developed, only a select few have sought to identify the optimal formulation for the successful encapsulation into PLGA nanoparticles. This FDA-approved polymer is highly biocompatible and biodegradable, making it a good candidate for pharmaceutical applications such as drug delivery [25]. We aimed to select key physio-chemical properties to construct an ideal nanoparticle in terms of drug release characteristics, cytotoxicity, and stability. The main characteristics we investigated were particle size, encapsulation efficiency, zeta potential, surface charge, and polydispersity index. These nanoparticles were fabricated using a modified double emulsion w1/o/w2 solvent evaporation method using ethyl alcohol as an organic solvent [26]. Data have shown that using ethyl alcohol produces stable nanoparticles, whereas other solvents including acetone, chloromethane, and dichloromethane failed to do so [27]. Polyvinyl alcohol was chosen as a stabilising agent for the second aqueous phase due to its ability to control the size of the formulation and produce particles with high encapsulation [28]. PVA biomaterials display high in vivo biocompatibility, low toxicity, and low biodegradability which is advantageous if the nanoparticles were administered intravenously. Particle size also is an important parameter to consider for cellular uptake, permeability, and avoiding rapid clearance. Some size limitations are known; very small nanoparticles of 10 nm are subject to renal clearance and phagocytosis, and therefore significantly reduce the accumulation of such particles at the target site [29]. Therefore, taking these into consideration, we constructed our nanoparticles to be within the recommended range of 10–1000 nm.

Rhodamine B, a fluorescent hydrophilic dye, was selected as a drug model since it was unable to penetrate the membrane of HEK293 cells when un-encapsulated yet, when loaded into nanoparticles, it could enter easily [30]. After 18 h, Rhodamine B-loaded PLGA nanoparticles were able to internalise into human endothelial cells and were visualised by TEM within the cystol. In comparison to other research, it appears that the rate at which PLGA nanoparticles are internalised is dependent upon incubation time, concentration of nanoparticles, and cell line. For instance, renal proximal epithelial cells were capable of internalising nanoparticles within 30 min, whereas colon Caco-2 epithelial cells only began uptake after 24 h [31]. The encapsulation efficiency of our vancomycin-loaded PLGA nanoparticles was optimised by altering the volume of inner aqueous phase, drug:polymer ratios, and stabiliser concentrations. Our final nanoparticles were capable of 82% *w*/*w* ± 12 vancomycin encapsulation, comparable to the highest value reported in the literature of 94.76% [32].

The in vitro efficacy model evaluated the ability of the formulated vancomycin nanoparticles to treat *S. aureus*-infected endothelial cells. As anticipated, cells that were treated with concentrated vancomycin solution showed reduced *S. aureus* growth, confirming the antibiotics’ ability to target extracellularly-bound bacterium. However, cells that were additionally supplemented with the vancomycin nanoparticles displayed significantly less *S. aureus* growth compared to cells only treated with vancomycin solution. This demonstrates the remarkable antimicrobial capability of the 10% vancomycin PLGA RG 503H nanoparticles, able to successfully target the internalised *S. aureus* compared to the application of free vancomycin alone.

The prevalence of persistent *S. aureus* infections is an enormous concern for survivors of sepsis or bacteraemia. We have found that *S. aureus* is capable of internalising into human endothelial cells through its surface expressed fibronectin binding proteins. This suggests that invasion of endothelial cells may be a virulence mechanism employed by *S. aureus* to evade circulating antibiotic treatment and cause recurrent infections. Anti-staphylococcal compounds like vancomycin are unable to internalise into cells, and therefore cannot target intracellular bacteria. Limitations surrounding current sepsis treatments can be overcome by coupling traditional antibiotics with drug-encapsulated nanoparticles to offer a significantly improved delivery system to the infection nidus. We have formulated 10% vancomycin HCl PLGA RG 503H nanoparticles that display 91% drug release in vitro, which were capable of significantly reducing bacterial growth within infected endothelial cells. These nanoparticles are highly efficient to counteract the virulent internalisation mechanisms employed by the bacterium and display future potential to target bacterial infections caused by *S. aureus*.

## 4. Materials and Methods

### 4.1. Bacterial Isolates and Cell Culture Conditions

The bacterial strains used in this study are *S. aureus* NCTC 8325-4 and isogenic mutant lacking the fibronectin binding proteins, *S. aureus* 8325-4 ΔfnbpAB. Both isolates were grown in Brain Heart Isolation (BHI) broth to mid-exponential growth phase at 37 °C (optical density 600 nm = 1). Human Aortic Endothelial Cells (HAoEC) (PromoCell) were maintained in Endothelial Cell Media supplemented with basal medium, penicillin (200 U/mL) and streptomycin (200 µg/mL).

### 4.2. Dot Blot Test

*S. aureus* isolates were cultured overnight as previously described and centrifuged at 3800 rpm for 7 min. After re-suspending the pellet in PBS, a 5 µL drop was placed onto a nitrocellulose membrane and allowed to dry at room temperature. The membrane was blocked for 20 min in 10% TBST (tris-buffered saline tween) followed by addition of a *S. aureus* FnBP primary antibody at 1:1000. Following washing, an HRB-donkey polyclonal secondary antibody was added at 1:10,000. The membrane was analysed for protein expression using an ECL detection kit.

### 4.3. Internalisation and Visualisation of S. aureus in Human Endothelial Cells

Human endothelial cells were seeded at a density of 5 × 104 cells per well and infected with *S. aureus* 8325-4 at a Multiplicity of Infection of 1000. After 1 h, cells were washed with PBS and lysed using 0.1% Triton X-100 to measure total bacteria. To measure intracellular bacteria, cells were washed and supplemented with 100 µg/mL gentamicin HCl for 1 h. Cells were lysed as described. Total bacterial counts were determined using colony forming units (CFU/mL). Internalised bacteria were visualised using Transmission Electron Microscopy (TEM). Briefly, infected endothelial cells were fixed in 2.5% glutaraldehyde, mounted on Pioloform copper coated grids, and imaged using a Hitachi H-7650 electron microscope. To confirm the re-emergence of internalised *S. aureus* after 0, 1, and 48 h of infection, cells were treated with gentamicin as described. The infected cells were lysed as described with 0.1% Triton X-100, and a 50 µL cell lysate sample was plated onto BHI agar to determine and quantify bacterial growth.

### 4.4. Preparation of Rhodamine B Nanoparticles

To confirm that the proposed formulation method (double-emulsion (water1-in-oil-in-water2) solvent evaporation) would encapsulate a hydrophilic molecule and result in nanoparticles suitable for cellular uptake in terms of size and physicochemical characteristics, the model hydrophilic drug Rhodamine B was first encapsulated. Rhodamine B is also fluorescent and therefore its location can be tracked intracellularly. Briefly, 0.5 mg Rhodamine B was dissolved in deionised water (1.0 mL) and emulsified dropwise with sonication (70%) into the PLGA-RG-503H polymer (99.5 mg dissolved in 4 mL ethyl acetate) in an ice bath. This primary emulsion was then emulsified by sonication (70%) into 10 mL PVA 2.5% *w*/*v* in an ice-bath. This mixture was poured into 35 mL PVA 1% (*w*/*v*) under magnetic stirring at 200 rpm for 16 h at room temperature. The nanoparticles were collected by ultracentrifugation at 11,000 rpm for 15 min at 20 °C. The pellet was washed three times in 20 mL deionized water and re-centrifuged to remove any residual PVA. Samples were resuspended in a cryoprotectant, 5% glucose (*w*/*v*) prior to long term storage by freeze drying. Nanoparticles intended for TEM imaging were resuspended in deionised water.

### 4.5. Preparation of Vancomycin HCl-Loaded Nanoparticles

Vancomycin HCl nanoparticles were first formulated using a base formulation. Briefly, 90mg of Vancomycin HCl was dissolved in deionised water (1.0 mL) and emulsified dropwise with sonication (70%) into the PLGA -RG-503H polymer (90 mg dissolved in 4 mL ethyl acetate) in an ice bath. This primary emulsion was then emulsified by sonication (70%) into 10 mL PVA 2.5% *w*/*v* in an ice-bath. This mixture was poured into 35 mL PVA 1% (*w*/*v*) under magnetic stirring at 200 rpm for 16 h at room temperature. The nanoparticles were collected by ultracentrifugation at 11,000 rpm for 15 min at 20 °C. The pellet was washed three times 20 mL deionized water and re-centrifuged. Samples were resuspended in a cryoprotectant, 5% glucose (*w*/*v*) prior to long term storage by freeze drying. Nanoparticles intended for TEM imaging were resuspended in deionised water. Subsequently, formulation parameters (drug:polymer ratio; volume of inner aqueous phase; PVA concentration) were varied to optimise the vancomycin HCl nanoparticle properties in terms of nanoparticle size, zeta potential and encapsulation efficiency. The optimal formulation parameters included a drug:polymer ratio of 1:9, an inner aqueous phase of 0.3 mL and PVA concentration of 2.5% *w*/*v*. Blank nanoparticles were formulated as per the base formulation, with the exception of the addition of Vancomycin HCl.

### 4.6. Immunofluorescence

Human endothelial cells were seeded onto glass coverslips at 3 × 105 cells then treated with 1 mg/mL Rhodamine B-loaded nanoparticles for 18 h. Cells were fixed using 4% formaldehyde and permeabilized with 0.1% Triton X-100. After blocking using 1% Bovine Serum Albumin (BSA), GAPDH 14C10 Rabbit mAb 1:50 was added followed by Alexa Fluor 647 goat anti-rabbit IgG 1:200. Coverslips were transferred to a glass slide with ProLong Diamond Antifade Mountant with DAPI then visualised using a Zeiss Axioscope for fluorescent imaging.

### 4.7. Characterising PLGA Nanoparticles

The average particle size, zeta potential, and polydispersity index (PDI) of either blank or loaded nanoparticles were analysed by photon correlation spectroscopy at 25 °C, using a Zetasizer Nano Series. The shape and surface morphology of the nanoparticles was observed by TEM on a silicon monoxide/formvar coated copper grid.

### 4.8. Determination of Encapsulation Efficiency

The encapsulation efficiency of nanoparticles was determined on freeze-dried samples. A weighted amount of sample was dissolved in acetonitrile and sonicated for 40 min. An equal quantity of water was added to precipitate the PLGA. The pellet was ultracentrifuged at 24,000 rpm for 20 min to pellet the precipitated PLGA. The supernatant was analysed using UV spectrophotometry at 280 nm to calculate drug loading per nanoparticle. The encapsulation efficiency (% *w*/*w*) was determined by the following equation: (amount of drug loaded/theoretical amount of drug loaded) × 100.

### 4.9. Nanoparticle Drug Release

To determine the amount of released vancomycin HCl from nanoparticles, in vitro release studies were performed using the PUR-A-LYZER mini 6000 dialysis kit. A dialysis bag containing a weighted amount of vancomycin PLGA nanoparticles in PBS at pH 7.4 was immersed in a shaking water bath at 100 rpm at 37 °C. At fixed time intervals, withdrawn samples were measured using UV spectrophotometry at 280 nm to quantify released vancomycin HCl.

### 4.10. Cytotoxic Evaluation of Formulation Nanoparticles

The MTT assay ((3-(4,5-dimethylthiazol-2-yl)-2,5-diphenyltetrazolium bromide) was used to examine cellular toxicity of the nanoparticles. Human endothelial cells were cultured at 2 × 105 cells/mL then treated for 24 h with 1 mg/mL PLGA nanoparticles. The viability of human endothelial cells was measured according to the MTT protocol followed by examination by UV spectrophotometry at 570 nm.

### 4.11. Pre-Clinical Evaluation of Formulated Nanoparticles

To examine whether vancomycin HCl PLGA nanoparticles were capable of treating human endothelial cells, they were infected with *S. aureus* 8325-4 as previously described and treated with 0.5 mg/mL vancomycin (10% *w*/*w*)-loaded nanoparticles for 18 h. After being lysed, the cell supernatant was plated onto BHI agar to calculate CFU/mL.

### 4.12. Statistical Analysis

Experiments were performed in independent triplicates, and statistically significant differences were analysed using one-way ANOVA followed by post hoc tests or *t*-tests using GraphPad Prism. Results were considered significant at *p* ≤ 0.05, and data was represented as mean ± standard error of mean (SEM).
